# Sirt1 Inhibits Oxidative Stress in Vascular Endothelial Cells

**DOI:** 10.1155/2017/7543973

**Published:** 2017-05-04

**Authors:** Weijin Zhang, Qiaobing Huang, Zhenhua Zeng, Jie Wu, Yaoyuan Zhang, Zhongqing Chen

**Affiliations:** ^1^Department of Critical Care Medicine, Nanfang Hospital, Southern Medical University, Guangzhou 510515, China; ^2^Guangdong Key Lab of Shock and Microcirculation Research, Department of Pathophysiology, Southern Medical University, Guangzhou 510515, China

## Abstract

The vascular endothelium is a layer of cells lining the inner surface of vessels, serving as a barrier that mediates microenvironment homeostasis. Deterioration of either the structure or function of endothelial cells (ECs) results in a variety of cardiovascular diseases. Previous studies have shown that reactive oxygen species (ROS) is a key factor that contributes to the impairment of ECs and the subsequent endothelial dysfunction. The longevity regulator Sirt1 is a NAD^+^-dependent deacetylase that has a potential antioxidative stress activity in vascular ECs. The mechanisms underlying the protective effects involve Sirt1/FOXOs, Sirt1/NF-*κ*B, Sirt1/NOX, Sirt1/SOD, and Sirt1/eNOs pathways. In this review, we summarize the most recent reports in this field to recapitulate the potent mechanisms involving the protective role of Sirt1 in oxidative stress and to highlight the beneficial effects of Sirt1 on cardiovascular functions.

## 1. Introduction

The vascular endothelium lining the inner walls of vessels has multiple functions such as maintaining microenvironment homeostasis, nutrient exchange, host defense reactions, and vasodilation [1]. Endothelial damage is secondary to a variety of stimuli and results in the loss of endothelial integrity, barrier dysfunction, and abnormal regulations of vasodilation and vasoconstriction, eventually leading to alteration of the vascular environment [2]. Subsequently, this alteration causes changes in vascular hemodynamics, affects organ perfusion, and results in the occurrence of cardiovascular events and a high incidence of mortality [3]. Therefore, great importance should be attached to protecting the structure and functions of microvascular endothelium. Reactive oxygen species (ROS) is produced in lots of pathogenesis, such as diabetic mellitus, asthma, and atherosclerosis, and ROS has been considered to play a pivotal role in endothelial dysfunction for decades [4–6]. When ECs are exposed to reactive oxygen, a subsequent endothelial hyperpermeability occurs and can result in various diseases such as acute respiratory disease syndrome and asthma, due to ROS-related cascade effects. Plasma extravasation and bronchial hyperresponsiveness were reported to occur after exposure to H_2_O_2_ inhalation [7]. Therefore, amelioration of ROS-dependent endothelial dysfunction represents a promising target to delay the development of related diseases.

**Figure 1:**
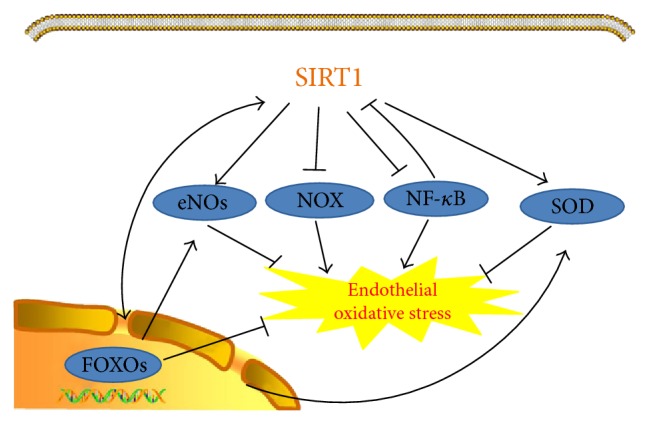
Signaling pathways of Sirt1 inhibiting reactive stress in ECs. There is interplay between Sirt1 and FOXOs in ROS reduction. Sirt1 also directly interacts with eNOs and SOD, which could be regulated through FOXOs. NOX and NF-*κ*B also serve as downstream target of Sirt1, which could be downregulated by NF-*κ*B activation. These molecules play pivotal roles in reactive stress resistance in ECs.

As a class III nicotinamide adenine dinucleotide- (NAD-) dependent histone deacetylase, Sirt1 has been demonstrated to regulate critical metabolic processes including oxidative stress, ageing, and apoptosis via deacetylation of a variety of substrates [8, 9]. Intriguingly, the inhibition of Sirt1 with pharmacological agents or siRNA [10] leads to an elevation of ROS levels, indicating a definite relationship between Sirt1 and ROS. However, the mechanisms underlying the Sirt1-mediated ROS decrease remain obscure. In this report, the interplay between Sirt1 and ROS will be elaborated. The signaling networks of Sirt1 involved in ROS resistance are shown in Figure 1.

## 2. Molecular Biology, Function of Sirt1

It is widely acknowledged that silencing information regulator complex (SIR complex) confers longevity for yeast [11]. In yeast, the SIR complex consists of four groups (Sir1-Sir4), among which Sir2 has been verified to be widespread in several types of cells [12]. In mammals, there are seven kinds of members (homologues of Sir2), which are termed sirtuins. According to molecular analysis of conserved core domain sequence of sirtuin from a variety of organs, the seven sirtuins are classified into 4 groups. Group I includes Sirt1, 2, and 3, group II includes Sirt4, group III includes Sirt5, and group IV includes Sirt6 and 7 [13]. Among these, Sirt1 is the most extensively studied homologue due to its similarity with Sir2 and its potential protective role in vascular disease. The gene encoding Sirt1 is located at 10q21.3, and length is 33715 bp, with nine exons encoding 747 amino acids, which includes 275 deacetylated amino acids located in the core domain [14].

So far, several natural and synthetic substances have been reported to activate Sirt1 and promote endothelial homeostasis. Resveratrol is the most widely used. Resveratrol is a subtype of phytoalexins that protects ECs against enhanced proinflammatory cytokines and reactive oxygen [15–17]. Resveratrol has been proven to induce mitochondrial biogenesis and promote vascular health [18]. Polydatin is another valuable ingredient extracted from the roots of the traditional Chinese herb called *Polygonum cuspidatum*. Our research team has validated that polydatin protects hepatocytes [19], small intestines [20], and kidney [21] against hemorrhagic shock by upregulating Sirt1 levels. Another active compound tetramethylpyrazine (TMP) is isolated from a Chinese herb and is capable of reversing high glucose-induced endothelial dysfunction via upregulation of Sirt1 [22]. Moreover, Vitamin D could remarkably reverse endothelial damage caused by oxidative stress, via Sirt1 activation [23]. Quercetin is a flavonol compound and inhibits oxidized LDL-induced EC damage, by activating Sirt1 [24]. In addition, there are several other naturally polyphenols, for instance, fisetin and butein that activate Sirt1 [25].

By interacting with several target proteins, Sirt1 exerts a wide range of cellular functions including energy balance, lipid homeostasis, and especially, endothelial protection from vascular diseases [26]. In aging arteries, downregulation of Sirt1 expression contributes to the formation of plaque and foam cells, which are related with atherosclerosis when endothelium is predisposed to attack. It has been shown that phosphorylation of Sirt1 at serine 47 induced by CDK5 activates Sirt1 and exerts antisenescence effect on vascular ageing [27]. Zhang et al. [28] suggested that Sirt1 expression should be controlled accurately for regulating metabolism homeostasis and inflammatory responses in order to delay or reverse the exacerbation of atherosclerosis. Mice deficient in Sirt1 had abnormal heart development and a severely shorten life span, indicating the pivotal role of Sirt1 in the maintenance of heart-protective functions. These effects of Sirt1 indicate that Sirt1 has a great potential to emerge as an attractive candidate for the amelioration of endothelial dysfunction [29]. Additionally, Sirt1 activation induced by pulsatile flow prevents EC dysfunction and retards the progression of atherosclerosis [30]. Furthermore, Sirt1 is also indispensable for the survival of cardiomyocytes, counteracting ischemia-reperfusion injury and cardiac rhythm [31, 32]. This review will discuss the molecular mechanisms of Sirt1 activation and the effect of Sirt1 on endothelial protection.

Functioning as an NAD^+^-dependent deacetylase, Sirt1 is capable of deacetylating numerous targets to protect ECs [33]. By deacetylating p53, Sirt1 may prevent stress-induced senescence and dysfunction of ECs [34]. Sirt1 could also promote proliferation and prolong senescence by targeting LKB1 in ECs [35]. Downregulation of p66Shc expression through Sirt1 activation protects vessels from hyperglycemia-induced EC dysfunction [36]. Furthermore, Sirt1 activator-induced mitochondrial biogenesis in ECs is mediated by the upregulation of targets such as proliferator-activated receptor gamma coactivator-1alpha (PGC-1*α*), nuclear respiratory factor 1 (NRF1), and eNOs [18]. Ghisays has reported that the N-terminal domain of Sirt1 enhanced its association with substrate NF-*κ*B p65 in the nucleus and decreased inflammation [37]. Increase of Sirt1 expression induced by resveratrol has been demonstrated to diminish TNF-*α*-induced CD40 elevation in human umbilical vein ECs (HUVECs) [16]. It is also widely acknowledged that Sirt1 deacetylase is an important in vivo regulator of autophagy [38]. Autophagy is activated in response to different kinds of stimuli by augmenting stress resistance and clearance ability [39, 40]. Resveratrol was reported to protect HUVECs from atherosclerosis by upregulating Sirt1 levels, restoring lysosomal function, enhancing autophagic flux, and accelerating Ox-LDL degradation through the autophagy-lysosome degradation pathway [15]. Taken together, the results of these studies help to elucidate the mechanisms by which Sirt1 exerts its protective effect on ECs.

## 3. Sirt1 Inhibits Oxidative Stress

Oxidative stress is characterized by the imbalance between the ROS production and oxidative stress resistance [41]. ROS includes superoxide (O_2_^−^), hydrogen peroxide (H_2_O_2_), hydroxide (OH^−^), and hypochlorite (OCl^−^). which participate crucially in the impairment of ECs. ROS plays a pivotal role in many diseases including atherosclerosis, diabetic mellitus, and myocardial dysfunction [42]. There is extensive interplay between reactive stress and Sirt1. Sirt1 has gained a lot of attention for its role in oxidative stress resistance. The mechanisms involved include Sirt1/FOXOs, Sirt1/NF-*κ*B, Sirt1/NOX, Sirt1/SOD, and Sirt1/eNOs pathways.

### 3.1. Sirt1 and FOXOs

FOXOs belong to a subgroup of Forkhead family of transcriptional factors. Invertebrates possess only one FOXO gene, whereas mammals have four FOXO genes: FOXO1, FOXO3, FOXO4, and FOXO6 [43]. In response to stress, FOXOs translocate into the nucleus and augment its protein expression, thus engaging in a variety of cellular functions that regulate cell cycle, enhance cell immunity, and inhibit oxidative stress. Evidence showed that activation of FOXO3 could protect quiescent cells from oxidative stress by directly binding to the manganese superoxide dismutase (MnSOD) promoter and enhancing expression of MnSOD to resist ROS [44]. Conversely, FOXO inhibition rendered a decrease in oxidative stress resistance and increase in ROS level, indicating the pivotal role of FOXOs in ROS resistance [45].

The link between FOXO and Sirt1 indicates an evolutionarily mechanism for oxidative stress resistance. FOXO1, FOXO3a, and FOXO4 are indispensable for Sirt1-dependent cell survival against oxidative stress [46]. It has been shown that Sirt1 and FOXO3 formed a complex upon stimulation with oxidative stress, and during both in vivo and in vitro conditions, Sirt1 deacetylated FOXO3 to induce resistance to oxidative stress [47]. Sirt1 enhances FOXO1 DNA binding ability by deacetylating FOXO1 and attenuates the oxidative stress response [48]. Similarly, Sirt1 binds to FOXO1 in an NAD-dependent manner and enables the accumulation of FOXO4 in the nucleus, producing DNA damage-inducible protein 45, a stress resistance-related gene [49]. However, the details of the interactions between FOXO1 and Sirt1 remain elusive for which one functions as upstream of the other. It was noted that FOXO3a modulated Sirt1 transcription by combing with p53 elements under nutrient stress, suggesting that FOXO3a might modulate Sirt1 expression [50]. Xiong et al. [51] indicated that FOXO1 directly activated Sirt1 expression in vascular smooth muscle cells and HEK293 cells. Overexpression of FOXO1 enhances Sirt1 levels, indicating that FOXO1 is a positive regulator of Sirt1. Intriguingly, Sirt1 activation can trigger FOXO1 deacetylation and augment FOXO-driven Sirt1 autotranscription. Therefore, autofeedback may participate in FOXO1-dependent Sirt1 transcription and Sirt1-mediated FOXO1 deacetylation.

In spite of the obscure interactions between Sirt1 and FOXO in response to oxidative stress, to a certain extent, their interaction with each other, indeed, benefits vascular ECs. Evidence showed that Sirt1-dependent activation of FOXO1 was crucial in vascular protection after the onset of oxidative stress [52]. Consistent with these results, a downregulation of Sirt1 and subsequent FOXO1-mediated reduction of mitochondrial antioxidant enzyme was induced by hyperglycemia, implying that Sirt1/FOXO1 axis might facilitate antioxidant effects in ECs [53]. In addition, Sirt1 and FOXO1 are crucial for angiogenesis and miR-217 can impair angiogenesis by inhibiting Sirt1 and FOXO1. In contrast, elevation of Sirt1 levels and FOXO1 deacetylation via inhibition of miR-217 in ECs increases antioxidant effects and prevents endothelial dysfunction [54]. Furthermore, Sirt1-deacetylated FOXO1 and subsequent repression of the antiangiogenic effects is considered to be a key factor contributing to the delayed senescence of ECs and a limited ROS accumulation in high glucose condition [55]. Therefore, crosstalk between Sirt1 and FOXO serves a crucial role in ROS resistance.

### 3.2. Sirt1 and NF-*κ*B

The transcriptional factor NF-*κ*B comprises of five subgroups including RelA or P65, RelB, c-Rel, p50/p105, and p52/p100, which remain quiescent because they are attached to an inhibitory protein I*κ*B. After IKK triggers I*κ*B phosphorylation and renders the degradation of I*κ*B, NF-*κ*B is activated and translocates from cytosol to the nucleus [56]. NF-*κ*B activation is closely related to reactive oxygen generation and greatly contributes to endothelial dysfunction. Several studies have revealed that antioxidant genes are under the control of NF-*κ*B. Through regulation of NF-*κ*B at the transcriptional level, a dramatic increase in mRNA levels for NF-*κ*B was detected and oxidative stress was induced after cadmium (Cd) exposure [57]. Moreover, oxidative stress could be induced through upregulation of ROS during pathogenesis of retinopathies and NF-*κ*B inhibitor SN50 dramatically reduces ROS production, indicating that oxidative stress is mediated by NF-*κ*B [58].

However, this process can be reversed through Sirt1 activation. Resveratrol protects HUVECs against TNF-*α*-induced injury through promoting Sirt1-induced repression of NF-*κ*B and ROS generation. The N-terminal domain of Sirt1 is a positive regulator of the endogenous Sirt1-dependent deacetylation capacity and promotes its physical association with NF-*κ*B p65 by deacetylating p65, thereby inhibiting NF-*κ*B transcription [16, 37]. In bovine retinal capillary endothelial cells (BRECs), Sirt1 overexpression inhibited the increase in mitochondrial reactive oxygen by diminishing NF-*κ*B expression while depletion of Sirt1 via siRNA presented reduced resistance to ROS induced by hyperglycemia stress [59]. It has been shown that vascular oxidative stress and NF-*κ*B were attenuated in response to caloric restriction (CR) in cultured coronary arterial endothelial cells (CAECs). Intriguingly, sera obtained from CR animals showed antioxidant effects and NF-*κ*B inactivation. The aforementioned effect was Sirt1 dependent [60]. Furthermore, NF-*κ*B and Sirt1 could be involved in antagonistic crosstalk during the regulation of ROS in ECs. Sirt1 inhibits NF-*κ*B by directly deacetylating the p65 subunit or activating AMPK and PPAR*α*, which inhibits the NF-*κ*B pathway and naturally suppresses ROS generation. Additionally, NF-*κ*B transcription suppresses Sirt1 activation through ROS production [61]. Taken together, the above findings indicate that Sirt1 regulates NF-*κ*B signaling and controls ROS attack, and NF-*κ*B itself could decrease Sirt1 levels to increase ROS production.

### 3.3. Sirt1 and NOX

NADPH oxidase (NOX) family consists of several members including: NOX isoforms, two organizer subunits (p47phox, NOXO1), two activator subunits (p67phox, NOXA1), and two DUOX-specific maturation factors (DUOXA1 and DUOXA2) [62]. The members function in multiple ways, including killing harmful microorganisms, regulating pH in the phagosome [63], transporting ions [64], and reducing inflammation. However, NOX can damage tissues and organs for their participation in ROS production after catalyzing their substrate molecules.

Sirt1 participates in diminishing NOX production. It was confirmed that Sirt1 inhibition was engaged in upregulation of NOX oxidase subunits, p22phox, and NOX4, eventually leading to endothelial dysfunction due to O_2_^−^ production [65, 66]. Meanwhile, quercetin-induced upregulation of Sirt1 enhances AMPK activity and decreases NADPH production, thus suppressing the hyperglycemia-induced oxidant damage in HUVECs. Therefore, the Sirt1/AMPK/NADPH pathway participates in antioxidant effects promoted by quercetin [24]. It has been shown that Sirt1 is a key player in cellular senescence and is NAD^+^-dependent. Decreases in NAD^+^ content induced by ROS tend to impair Sirt1 activity [67]. However, increased activity of NOX may enhance NAD^+^ content and Sirt1 levels to induce oxidized state in ECs. These effects were explained by the moderate and transient increase in ROS, which induced Sirt1 expression, inconsistent with the findings that short-term H_2_O_2_ treatment could enhance Sirt1 levels [68, 69]. Hence, an agonistic relationship exists between NOX and Sirt1 at low dose of reactive oxygen.

### 3.4. Sirt1 and SOD

SOD (superoxide dismutase), a group of metal-containing enzymes, is characterized by their ability to scavenge reactive oxygen species. SOD enzymes comprise of 3 members, including SOD1 (cytoplasmic), SOD2 (mitochondrial), and SOD3 (extracellular). SODs have a pivotal role in oxidative stress resistance specifically in liberating H_2_O_2_ by binding to a superoxide anion with their metal zipper. Deficiency or impairment of metals in SODs including Cu-SOD, Zn-SOD, Ni-SOD, Mn-SOD, and Fe-SOD contributes to oxidative stress directly [41].

Sirt1 has been shown to promote manganese superoxide dismutase (MnSOD) expression and increase the oxidative stress resistance in human retinal microvascular endothelial cell (RMECs). Sirt1 potentiates FOXO3a activation by deacetylating FOXO3a, which increases the transcription of downstream genes such as MnSOD. Therefore, Sirt1/FOXO/MnSOD may contribute to oxidative stress resistance in ECs [70]. Moreover, elevation of NAD^+^ levels and Sirt1 expression was detected after nicotinamide mononucleotide (NMN) treatment in thoracic aorta. These effects were accompanied by MnSOD enhancement, which was probably modulated by Sirt1 to exert vascular antioxidant effect [71]. Rapidly increasing prevalence of diabetic mellitus has been posing great threat to public health worldwide because of the resulting endothelial dysfunction induced by oxidative stress. Studies have shown that MnSOD was significantly downregulated in the aortas of diabetic WT mice, whereas endothelium-specific Sirt1 transgenic mice successfully reversed the MnSOD decline, thereby indicating the pivotal role of the Sirt1/MnSOD pathway in the inhibition of hyperglycemia-induced endothelial dysfunction [72]. Similarly, MnSOD expression was elevated by resveratrol and conferred antioxidative stress protection in CAECs, which was diminished by Sirt1 knockdown and mimicked by Sirt1 overexpression [17]. Therefore, the involvement of Sirt1 activation and subsequent SOD upregulation may ameliorate endothelial oxidative stress.

### 3.5. Sirt1 and Endothelial Nitric Oxide Synthase (eNOs)

NOs families comprise of three groups including neuronal nitric oxide synthase (nNOs) expressed in vascular smooth muscle, inducible nitric oxide synthase (iNOs) present in blood vessels under abnormal conditions, and endothelial nitric oxide synthase (eNOs) prominently expressed in ECs [73]. eNOs makes great contributions to oxidative stress resistance by producing nitric oxide (NO) and inhibiting O_2_^−^ generation [74]. The functions of eNOs are greatly dependent of its cysteine residues. Under oxidative stress, S-glutathionylation of eNOs occurs, accompanied by a decrease in NO activity and an increase in O_2_^−^ generation. Therefore, oxidative stress could abolish eNOs activity through S-glutathionylation [75].

The relationship between Sirt1 and eNOs in the process of defending oxidative stress has been reported extensively. It was demonstrated that Sirt1 agonist SRT1720 exerted salutary effects on expression of eNOs thus protecting HUVECs from senescence induced by H_2_O_2_. Furthermore, Sirt1 has been shown to promote endothelium-dependent vasodilation by targeting eNOs for deacetylation. Sirt1 and eNOs colocalize in ECs, and subsequently, Sirt1 activates eNOs through deacetylating lysine 496 and 506, and as a result, NO production is increased. Consequently, its ability for antioxidant stress is enhanced because NO bioavailability is closely related to increased oxidative stress resistance [76, 77]. Consistently, NO bioavailability was also verified to be increased and was dependent on Sirt1-deacetylated eNOs after pretreatment with docosahexaenoic acid [78]. In addition to deacetylating eNOs, Sirt1 has been demonstrated to play a pivotal role in eNOs phosphorylation. Inhibition of Sirt1 with Sirt1 siRNA decreased eNOs phosphorylation and abolished the protective effects of curcum on H_2_O_2_-induced senescence in HUVECs, suggesting the phosphorylation of eNOs by Sirt1 is a promising strategy for combating premature senescence of HUVECs [79]. Intriguingly, Sirt1 was not absolutely the direct upstream of eNOs. Evidence showed that Sirt1 could activate FOXOs to synthesize antioxidants, and inhibition of Sirt1, FOXO1, and FOXO3 attenuated the effects of resveratrol on eNOs activation, indicating that the Sirt1/FOXO axis is responsible for eNOs elevation [80]. In addition to the Sirt1/FOXO pathway, eNOs and NO are produced and are induced in a Sirt1/Krüpple link factor 2 (KLF2)-dependent manner and regulate endothelial function [81]. Therefore, the interplay between Sirt1 and eNOs serves as a salutary role in ROS resistance.

Although Sirt1 may be considered to be an optimal therapeutic target for vascular diseases, discreet evaluations should be conducted regarding the dosage to be used for therapy. As reported, 2.5–7.5-fold overexpression of Sirt1 prevents heart from oxidative stress via Sirt1/FOXO axis accompanied by the consumption of NAD^+^. If NAD^+^ is overconsumed due to higher levels of Sirt1, mitochondrial biogenesis reduces and its stress resistance is reversed, suggesting that the beneficial effect of Sirt1 can only be achieved at low to moderate doses. Careful evaluation is needed to determine therapeutic doses in clinical practice [4].

## 4. Conclusion

ROS causes progressive deterioration of the structure and function of ECs. As an antioxidative stress molecule, Sirt1 has been identified and studied to determine its role in ROS resistance in ECs. Intriguingly, transient increase of ROS could induce Sirt1 which, in turn, causes a decrease in ROS. However, high ROS levels diminish Sirt1 activation. The explanation for these effects may involve different cellular activities to counteract ROS. There is a complex signaling network for Sirt1-mediated ROS reduction involving Sirt1/FOXOs, Sirt1/NF-*κ*B, Sirt1/NOX, Sirt1/SOD, and Sirt1/eNOs. Among these factors, eNOs and SOD are downstream of FOXO, which could also activate Sirt1 and trigger the signaling pathway through positive feedback. Therefore, the positive network of the several antioxidant molecules would amplify the effect to defend oxidative stress. However, Sirt1 also serves as a key target of NF-*κ*B, whose transcription suppresses Sirt1 activation, resulting in the increase in ROS generation and a decrease in ROS scavenging. Furthermore, antagonist effect exists between SOD and NF-*κ*B. Transactivator of transcription (Tat)-SOD protein can hinder NF-*κ*B activation, defending the oxidation-driven atherosclerosis in HUVECs [82]. Together with this, SOD could also attenuate NF-*κ*B and reverse monolayer hyperpermeability induced by release of hemoglobin with hemolysis [83]. Activation of NF-*κ*B was suppressed by NOX inhibition in ECs, indicating the interplay between NF-*κ*B and NOX [84]. The NOX/NF-*κ*B signaling was also shown to engage in aggravated endothelial dysfunction due to high-dose intravenous iron supplementation [85]. It has been shown that NOX2-p47phox complex is formed to activate eNOs phosphorylation and NO production in ECs exposed to laminar shear stress, whereas the NOX1-NOXO1 complex could uncouple eNOs in the condition of atherogenic oscillatory shear stress, thus injuring ECs [86]. Probably, different submits of NOX may exert different effects on ECs by interacting with eNOs. Therefore, the downstream molecules of Sirt1 may interact with each other as a network, to amplify the antioxidant effect.

Despite the promising evidence, however, due to the fact that excessive expression of Sirt1 seems to exert the opposite effect, appropriate upregulation of Sirt1 may be taken into consideration for vascular disease therapy. Therefore, much more studies in high quality should be carried out for Sirt1 elevation regarding the optimal dosage as therapeutic use and its practical potential for disease prevention and protection in clinical application.
